# Effects of Polyunsaturated Fatty Acids Supplementation on the Meat Quality of Pigs: A Meta-Analysis

**DOI:** 10.3389/fnut.2021.746765

**Published:** 2021-09-29

**Authors:** Liyi Wang, Yuqin Huang, Yizhen Wang, Tizhong Shan

**Affiliations:** ^1^College of Animal Sciences, Zhejiang University, Hangzhou, China; ^2^Key Laboratory of Molecular Animal Nutrition (Zhejiang University), Ministry of Education, Hangzhou, China; ^3^Key Laboratory of Animal Feed and Nutrition of Zhejiang Province, Hangzhou, China

**Keywords:** polyunsaturated fatty acids, meat quality, pig, meta-analysis, conjugated linoleic acid, linseed

## Abstract

Polyunsaturated fatty acids (PUFAs) supplementation has been widely discussed as a strategy for improving meat quality in pig production, but the effects are inconsistent. This meta-analysis was performed to comprehensively evaluate its effects on the meat quality and growth performance of pigs. We searched the PubMed and the Web of Science databases (articles published from January 1, 2000 to October 16, 2020) and compared PUFAs-supplemented diets with control diets. We identified 1,670 studies, of which 14 (with data for 752 pigs) were included in our meta-analysis. The subgroup analysis was classified as PUFA source [conjugated linoleic acid (CLA) or linseed], concentration (high or low concentration), and initial stage (growing or finishing pigs). Our analysis found that PUFA supplementation increased the intramuscular fat (IMF) content (WMD = 0.467%, 95% CI: 0.312–0.621, *p* < 0.001), decreased the meat color L^*^ (WMD = −0.636, 95% CI: −1.225 to −0.047, *p* = 0.034), and pH 24 h (WMD = −0.021, 95% CI: −0.032 to −0.009, *p* < 0.001) but had no influence on drip loss, meat color a^*^ and b^*^, pH 45 min, and growth performance. CLA supplementation improved IMF content (WMD = 0.542%, 95% CI: 0.343–0.741, *p* < 0.001) and reduced meat color b^*^ (WMD = −0.194, 95% CI: −0.344 to −0.044, *p* = 0.011). Linseed supplementation increased IMF content (WMD = 0.307%, 95% CI: 0.047–0.566, *p* = 0.021), decreased meat color L^*^ (WMD = −1.740, 95% CI: −3.267 to −0.213, *p* = 0.026), and pH 24 h (WMD = 0.034, 95% CI: −0.049 to −0.018, *p* < 0.001). We discovered an increase on the IMF content in both high and low concentration PUFA supplementation (WMD = 0.461%, 95% CI: −0.344 to −0.044, *p* < 0.001; WMD = 0.456%, 95% CI: 0.276–0.635, *p* < 0.001). Furthermore, we also found the effects of PUFA supplementation on meat color L^*^ and pH 24 h are concentration- and stage-dependent. PUFA supplementation can improve the meat quality of pigs, which mainly emerges in greatly increasing IMF content.

## Introduction

There has been an increased interest in recent years in ways to produce high-quality pork. This is because pork is one of the most consumed animal proteins in the world and is an important source of dietary protein and fatty acid, especially saturated fatty acids, which is closely related to human health (1). The main traits by which pork quality is evaluated include intramuscular fat (IMF) content, drip loss, meat color, pH, juiciness, tenderness, flavor, and fatty acid composition. IMF is mainly distributed in the epimysium, perimysium, and endomysium of skeletal muscle and is positively correlated with meat quality including flavor, tenderness, and juiciness (2). Multiple factors can influence pork quality, such as nutrition, genetics, environment, management practices, and production systems (3); hence, it is of great significance to improve pork quality *via* seeking effective strategies.

Dietary intervention is one of the most common methods to improve the meat quality of pigs. Previous studies have found that dietary fatty acid composition plays an important role in regulating the nutritional quality of pork not only in lean pig breeds but also in Chinese indigenous breeds pigs (4, 5). It is a consumer-acceptable and effective strategy for producers to improve the meat quality of pigs through added fatty acid supplementation in diet. Polyunsaturated fatty acids (PUFAs) are one of the essential fatty acids, including n-3 PUFAs and n-6 PUFAs. PUFAs play an irreplaceable role in regulating fat deposition, muscle development, and glycolipid metabolism (6–8). In recent years, many studies have conducted feeding trials on pigs to explore the effects of PUFAs on meat quality with inconsistent results. The most commonly used PUFA supplementation is conjugated linoleic acid (CLA) and linseed. CLA is a secondary derivative of linoleic acid, and linseed is the ripe seed of flax. Several factors lending to these treatment effect inconsistencies include several factors, such as different PUFA source supplementation (CLA or linseed), added concentration (high concentration or low concentration), and initial growth stage of pigs (growing or finishing pigs), led to the inconsistent results by further comparison.

The aim of our study was to reveal the main effects or the effect orientation of PUFA supplementation on the meat quality of pigs by performing a meta-analysis. We also elucidated the potential influential factors based on the outcomes including IMF content, drip loss, meat color, pH 45 min, and pH 24 h. This is the first comprehensive and systematic meta-analysis focused on this topic and providing useful strategies for producing high-quality pork in the pig industry.

## Materials and Methods

We conducted and reported the meta-analysis strictly following the Preferred Reporting Items for Systematic Reviews and Meta-Analyses (PRISMA) Statement (9).

### Search Strategy

We collected studies from the last 20 years published between January 1, 2000 and October 16, 2020 in the PubMed (https://www.thncbi.nlm.nih.gov/pubmed; accessed October 16, 2020) and Web of Science (http://webofknowledge.com; accessed October 16, 2020) databases. We applied no language restrictions. The complete search principles were as follows: (1) the term “pigs” was searched in the PubMed database beforehand and shown to be “swine,” “suidae,” “warthogs,” “wart hogs,” “hog, wart,” “hogs, wart,” “wart hog,” and “phacochoerus”; (2) similarly, the terms related to PUFAs were extended to include “fatty acids, unsaturated,” “acids, unsaturated fatty,” “unsaturated fatty acids,” “acids, unsaturated fatty,” and “fatty acids, polyunsaturated”; and (3) meat quality was equal to pork quality and meat characteristic. The detailed search strategy and findings are shown in Table 1. We considered all potentially eligible studies instead of the primary outcome or language. We also did a manual search to obtain more studies. The complete search method was shown in Table 1.

**Table 1:** Search strategy.

**Search**	**Query**	**Items found**
**PubMed**		
#1	Search: (((((((((Swine) or Suidae) or Pigs) or Warthogs) or Wart Hogs) or Hog, Wart) or Hogs, Wart) or Wart Hog) or Phacochoerus)); Filters: Publication date from 2000/01/01 to 2020/10/16	157,481
#2	Search: ((((((Fatty Acids, Unsaturated) or Acids, Unsaturated Fatty) or Unsaturated Fatty Acids) or Polyunsaturated Fatty Acids) or Acids, Polyunsaturated Fatty) or Fatty Acids, Polyunsaturated))	122,686
#3	Search: (((meat quality) or pork quality) or meat characteristic))	18,919
#1 AND #2 AND #3		274
**Web of science**		
#1	TS = (Swine or Suidae or Pigs or Warthogs or Wart Hogs or Hog, Wart or Hogs, Wart or Wart Hog or Phacochoerus)	411,441
#2	TS = (Fatty Acids, Unsaturated or Acids, Unsaturated Fatty or Unsaturated Fatty Acids or Polyunsaturated Fatty Acids or Acids, Polyunsaturated Fatty or Fatty Acids, Polyunsaturated)	102,584
#3	TS= (meat quality or pork quality or meat characteristic)	117,463
#1 AND #2 AND #3		1396

### Selection Criteria and Procedure

**Figure 1:**
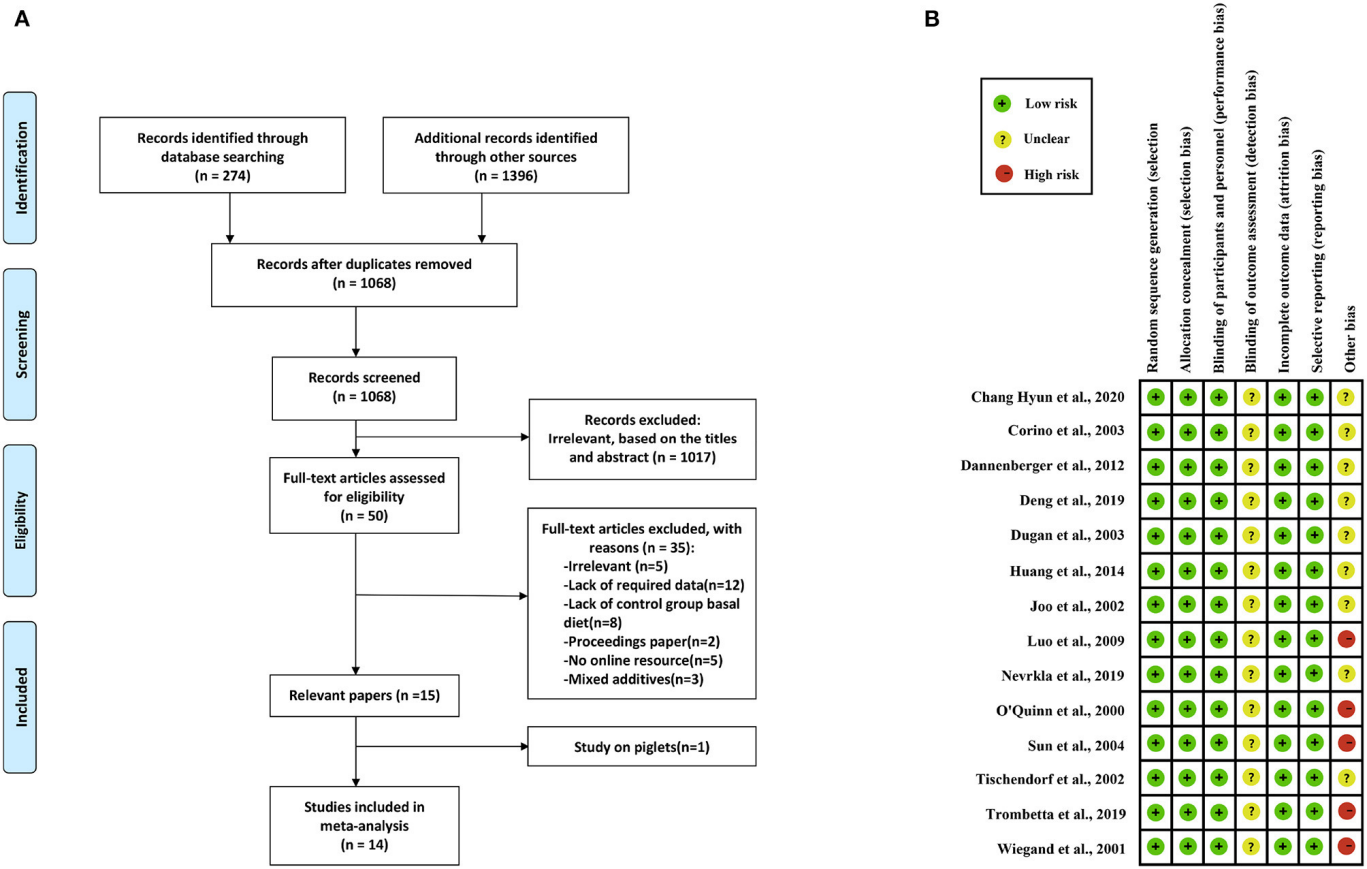
Study selection process and quality assessment. **(A)** Flowchart for the study selection process. **(B)** Study quality assessment.

We regarded studies as eligible for inclusion if they met the following criteria: (1) studies reported the effects of PUFAs on meat quality (IMF, drip loss, meat color, pH 45 min, and pH 24 h); (2) PUFAs, PUFA-rich compounds, PUFA supplements, or PUFA extracts were added to the feed throughout the experimental period; (3) the growth stage of pigs was growing or finishing; and (4) the concentration of PUFA supplements was reported. The exclusion criteria were as follows: (1) studies lacked a control group; (2) studies are proceedings papers; (3) studies lacked full-text online resources; (4) studies used mixed additives; and (5) studies investigated piglets. Based on these criteria, we screened eligible studies for subsequent meta-analysis (Figure 1A).

The following information was extracted from each selected study: author information (first author, year, and country), genetic background, PUFA source, experimental duration, added concentration, basal diet, energy difference, sum number of pigs included in the control and treatment groups, sex, growth stage (growing, finishing, or growing-finishing), growth performance parameters [average daily gain (ADG), average daily feed intake (ADFI), and gain:feed (G:F) ratio], and outcomes of meat quality (IMF, drip loss, meat color, pH 45 min, and pH 24 h). One study might have more than one record due to the duration of the pigs and concentration of the supplemental substance.

The study selection procedure was as follow: (1) two investigators (L. Wang and Y. Huang) independently screened the titles and abstracts of the articles according to the inclusion criteria; (2) disagreements during independent selection were solved through consultation with a third author (T. Shan); and (3) after the included studies were verified and confirmed, one investigator (L. Wang) extracted the data and information from each study and the other investigator (Y. Huang) checked. The summarized information of included studies was shown in Table 2.

**Table 2:** Characteristics of included studies^a^.

**Refernces**	**Country**	**Genetic background**	**PUFA source**	**Duration**	**Concentration**	**Basal** **diet**	**Energy difference^b^**	**N^c^**	**Sex**	**Growth stage**	**Growth performance parameters^d^**	**Outcomes^e^**
O'Quinn et al. (10)	American	PIC L326 or 327 boars × C22 sows	CLA 60	37.6–106.4 kg	50%	Corn-soybean	NA	24	Barrows	Growing-finishing pigs	ADG, ADFI, G:F ratio	Drip loss, meat color
Wiegand et al. (11)	American	NA	Conjugated linoleic acid	40–106 kg	0.75%	Corn-soybean	→	20	Barrows	Growing-finishing pigs	ADG, ADFI, G:F ratio	IMF, meat color
Joo et al. (12)	Korea	Landrace × Large White × Duroc	Conjugated linoleic acid	4 weeks	1%, 2.5%, 5%	NA	NA	20	Gilts	Finishing pigs	NA	IMF, drip loss, meat color, pH 24 h
Tischendorf et al. (13)	Germany	Pietrain × (Landrace × Large White)	Conjugated linoleic acid	8 weeks	2%	Barley-soybean	→	40	20 female and 20 male- castrated	Growing-finishing pigs	ADG, ADFI, G:F ratio	IMF, drip loss, meat color, pH 45 min, pH 24 h
Corino et al. (14)	Italy	Large White	Conjugated linoleic acid	97–172 kg	0.25%, 0.5%	Corn-soybean	→	36	18 barrows and 18 gilts	Finishing pigs	NA	meat color, pH 45 min, pH 24 h
Dugan et al. (15)	Canada	NA	Conjugated linoleic acid	35–115 kg	0.25%, 0.5%	Barley-soybean	→	108	NA	Growing-finishing pigs	NA	IMF, drip loss, meat color, pH 24 h
Sun et al. (16)	China	Duroc × Landrace × Large White	Conjugated linoleic acid	3, 6 weeks	2%, 4%	Corn-soybean	→	54	Crossbred barrows	Finishing pigs	ADG, ADFI, G:F ratio	IMF, drip loss
Luo et al. (17)	China	Landrace × NewDamLine	Linseed	30, 60, 90 days	10%	Corn-soybean	→	24	Barrows	Growing-finishing pigs	NA	IMF, drip loss, pH 45 min
Dannenberger et al. (18)	Germany	Landrace	High, reduced protein diet with linseed oil	60~100 kg to 120 kg	4.5%	Barley-soybean	↑	24	Male-castrated	Finishing pigs	ADG, ADFI, G:F ratio	IMF, drip loss, meat color, pH 45 min, pH 24 h
Huang et al. (19)	China	Rongchang pigs	Conjugated linoleic acid	30–60 kg, 60–90 kg	0.5%, 1%, 1.5%, 2%	Corn-soybean	→	160	NA	Growing-finishing pigs	NA	IMF, meat color, pH 45 min, pH 24 h
Deng et al. (20)	China	NA	Flaxseed	72 days	5%, 10%	Corn-soybean	→	72	NA	Growing-finishing pigs	ADG, ADFI, G:F ratio	IMF, drip loss, meat color, pH 45 min, pH 24 h
Nevrkla and Vaclavkova (21)	Czech Republic	(Large White × Landrace) × (Duroc × Pietrain)	Linseed	57 days	7%	Barley-soybean	↑	40	Gilts	Finishing pigs	NA	IMF, drip loss, pH 45 min, pH 24 h
Trombetta et al. (22)	Brazil	50% Large White ×50% Landrace	Linseed oil	90 days	3%	Corn-soybean	NA	22	10 castrated males and 12 females	Finishing pigs	ADG	IMF, drip loss, meat color, pH 24 h
Chang Hyun et al. (23)	Korea	Landrace × Yorkshire × Duroc	Linseed (n-6: n-3 PUFA ratio)	NA	1.5% (4:1), 3% (2:1)	Corn-soybean	→	108	NA	Finishing pigs	ADG, ADFI, G:F ratio	Drip loss, meat color, pH 45 min, pH 24 h

a *NA, not available*.

b *↑, higher energy density in treatment group; →, similar energy density in treatment and control groups; ↓, lower energy density in treatment group*.

c *Number of pigs included in studies*.

d *ADG, average daily gain; ADFI, average daily feed intake; G: F ratio, gain: feed ratio*.

e *IMF, intramuscular fat; pH 45 min, pH value measured at 45 min postmortem; pH 24 h, pH value measured at 24-h postmortem*.

### Study Quality Assessment

Two investigators (L. Wang and Y. Huang) independently assessed the quality assessment of included studies by using two methods: the Cochrane Handbook for Systematic Review of Interventions (24) and the Study Quality Assessment on Nonruminants (SQANR) (25), which is a new assessment method for feeding trials and included five detailed part: within-group differences, multiple reports, sample size, rationality of experimental design, and completeness of experimental information. Articles were judged as high risk, low risk, or unclear in the following aspects: random sequence generation, allocation concealment, blinding of participants and personnel, blinding of outcome assessment, incomplete outcome data, selective reporting, and other bias, in which the assessment of other bias according to the final score of SQANR (high and moderate qualities were defined as unclear risk, and low quality was defined as high risk) (Figure 1B and Supplementary Table 2).

### Within-Group Standard Deviation Estimate

We obtained the within-group SD by the following three approaches: (1) used the within-group SE to calculate; (2) contacted the authors *via* emails if the study has neither the within-group SD nor SE; and (3) used pooled SD as within-group SD, which was calculated from SEM, and pooled SD is equal to SEM multiplied by the square root of the number of groups (26).

### Statistical Analysis

The statistical analysis was performed with Stata 15.1 (Stata Corp., College Station, TX, USA).

#### Meta-Analysis

For continuous outcomes, because the units of measure data are the same and the mean varies little, we used a random-effects model to calculate the overall effect as weighted mean difference (WMD) and 95% CI between the treatment and control groups. If the 95% CI contained a zero value, there was no difference. We also used Cochran's *Q*-test (significance level of *p* ≤ 0.1) and the *I*^2^ statistic to assess the degree of statistical heterogeneity among studies, with a value of <25, 25–50%, 51–75, and >75% considered as no, low, moderate, and high level of heterogeneity, respectively (27). Particularly, based on Cochrane Handbook Chapter 10, we changed into fixed-effects model to meta-analysis if we found the statistics of study have homogeneity which meant *I*^2^ statistic <50%.

#### Regression Analysis

We performed a meta-regression analysis to explore the potential sources of heterogeneity and define the effects of covariates on outcomes (IMF, drip loss, meat color, pH 45 min, and pH 24 h) (28). The covariates were as follows: PUFA source (CLA or linseed), added concentration [high concentration (>2%) or low concentration (≤ 2%)], and initial growth stage (growing pigs or finishing pigs). The regression analysis was applied only to groups with 10 or more records to avoid a false positive result.

#### Subgroup Analysis and Sensitivity Analysis

To explore the sources of heterogeneity, we conducted a subgroup analysis if the study was regarded as having moderate or high heterogeneity (*I*^2^ > 50%). We classified the subgroups into three groups: CLA group or linseed group, high concentration group or low concentration group, and growing pigs group or finishing pigs group and foreign pigs or Chinese local pigs. If the heterogeneity was significant (*p* < 0.05), we also performed a sensitivity analysis to identify which study (or studies) contributing to the heterogeneity using the leave-one-out method. Heterogeneity and pooled analyses were recalculated after a single study was removed from the outcome at a time. We included data which the source of heterogeneity was identified and exclude these data did not influence the pooled estimates.

#### Publication Bias

The potential publication bias was investigated by funnel plot asymmetry (Supplementary Figure 1), Begg's and Egger's weighted regression test, for which the significance level was defined at *p* < 0.05 (29). We used Egger's test as a reference if funnel plot asymmetry, Begg's and Egger's tests disagreed. In addition, the trim-and-fill test was used to estimate the effect of publication bias on the interpretation of the results (30).

## Results

We identified 1,670 studies, of which 14 (with data for 752 pigs) were included in our meta-analysis (Figure 1A) (10–23). The 14 studies were all published between 2000 and 2020, and there was no repetition between studies (Table 1). These studies investigated the effects of PUFA supplementation on meat quality (IMF, drip loss, meat color, pH 45 min, and pH 24 h) and growth performance (ADG, ADFI, and G:F ratio). Among the selected studies, there are eight added CLA and six added linseed or linseed oil, seven studies (14 records) began at the grower phase, and seven studies (17 records) began at the finisher phase. The study quality assessment was shown in Figure 1B. We defined the risk of detection bias as unclear because the blinding of outcome assessment was not reported in the included studies. Other bias were assessed based on the final score of SQANR (Supplementary Table 2); there are nine studies: four have unclear risk and five have high risk. According to the funnel plot (Supplementary Figure 1), Begg's and Egger's tests, the publication bias was not significant (*p* > 0.05) in the current meta-analysis (Table 3), so the trim-and-fill test was not necessary to perform.

**Table 3:** The summary of meta-analysis and publication bias analysis of the included studies.

**Outcome^a^**	**N^b^**	**WMD (95% CI)^c^**	***P***	***I^2^***	***P***_**heterogeneity**_	**Begg's Test**	**Egger's test**
IMF (%)	26	0.467 (0.312–0.621)	<0.001	87.0%	<0.001	0.005	0.085
Drip loss (%)	24	−0.191 (−0.458 to 0.075)	0.159	63.4%	<0.001	0.861	0.439
L*	25	−0.636 (−1.225 to −0.047)	0.034	65.5%	<0.001	0.110	0.509
a*	25	0.081 (−0.244 to 0.406)	0.625	67.5%	<0.001	1.000	0.614
b*	25	−0.123 (−0.268 to 0.022)	0.095	53.4%	0.001	0.158	0.136
pH 45 min	24	0.038 (−0.042 to 0.117)	0.351	71.9%	<0.001	0.053	0.114
pH 24 h	24	−0.021 (−0.032 to −0.009)	<0.001	11.6%	0.300	0.516	0.229

a L^*^, lightness, a^*^, redness, b^*^, yellowness.

b *N, number of comparisons*.

C *WMD, weighted mean difference; CI, confidence interval*.

### Effects of PUFA Supplementation on the Meat Quality and Growth Performance of Pigs

As shown in Table 3, we presented the effects of PUFA supplementation on the meat quality of pigs. PUFA supplementation increased the content of IMF by 0.467% (95% CI: 0.312–0.621, *p* < 0.001) with high heterogeneity (*I*^2^ = 87.0%, *p*_heterogeneity_ < 0.001), decreased the meat color L^*^ by 0.636 (95% CI: −1.225 to −0.047, *p* = 0.034) with moderate heterogeneity (*I*^2^ = 65.5%, *p*_heterogeneity_ < 0.001) and decreased the pH 24 h by 0.021 (95% CI: −0.032 to −0.009, *p* < 0.001) with no heterogeneity (*I*^2^ = 11.6%, *p*_heterogeneity_ = 0.300). However, PUFA supplementation had no effect on the drip loss (WMD = −0.191, 95% CI: −0.458 to 0.075, *p* = 0.159) with moderate heterogeneity (*I*^2^ = 63.4%, *p*_heterogeneity_ < 0.001), meat color a^*^ (WMD = 0.081, 95% CI: −0.244 to 0.406, *p* = 0.625) with moderate heterogeneity (*I*^2^ = 67.5%, *p*_heterogeneity_ < 0.001), meat color b^*^ (WMD = −0.123, 95% CI: −0.268 to 0.022, *p* = 0.095) with moderate heterogeneity (*I*^2^ = 53.4%, *p*_heterogeneity_ = 0.001), and pH 45 min (WMD = 0.038, 95% CI: −0.042 to 0.117, *p* = 0.351) with moderate heterogeneity (*I*^2^ = 71.9%, *p*_heterogeneity_ < 0.001). Furthermore, we also presented the effects of PUFA supplementation on the growth performance of pigs in Supplementary Table 3. We found there is no significant difference in ADG, ADFI, and G:F ratio between the control and the PUFA supplementation group (*p* > 0.05).

### Regression Analysis and Sources of Heterogeneity

To explore the potential sources of heterogeneity and define the effects of covariates on meat quality and growth performance, we performed a meta-regression analysis (Table 4 and Supplementary Table 4). We found PUFA source, added concentration, and initial growth stage might play an important role in affecting the meat quality and growth performance, especially IMF content, because *p*_regression_ was 0.020, 0.021, and 0.006, respectively. Therefore, we performed subgroup analysis of PUFA source, added concentration, initial growth stage, and breeds in the subsequent research to explore detailed sources of heterogeneity (Table 4, Supplementary Tables 4, 5). We figured out the significant heterogeneity of drip loss and L^*^ came from linseed, high concentration, finishing pigs, and foreign pigs subgroup. CLA, low concentration, finishing pigs, and foreign pigs subgroup are sources of b^*^ heterogeneity. The PUFA source and breeds are the source of a^*^, an initial growth stage is the source of pH 45 min, and the concentration is the source of ADFI. We used sensitivity analysis through the leave-one-out method to explore the heterogeneity in IMF as we did not find the source of IMF heterogeneity according to the subgroup analysis. The significant heterogeneity had no alteration after each included study was removed, so we assume that the meta-analysis results are robust and the heterogeneity did not interfere with the direction and significance of the final results.

**Table 4:** Regression and subgroup analysis of studies included in the meta-analysis.

**Outcome**	**Subgroup**		**N^a^**	** $P_{\text{regression}}^{\text{b}}$ **	**WMD (95% CI)**	***P***	***I^2^***	***P***_**heterogeneity**_
IMF (%)	PUFA source	CLA	193	0.020	0.542 (0.343–0.741)	<0.001	86.4%	<0.001
		Linseed	81		0.307 (0.047–0.566)	0.021	86.6%	<0.001
	Concentration	High concentration	97	0.021	0.461 (0.208–0.715)	<0.001	92.2%	<0.001
		Low concentration	177		0.456 (0.276–0.635)	<0.001	72.0%	<0.001
	Initial growth stage	Growing pigs	200	0.006	0.563 (0.395–0.731)	<0.001	75.7%	<0.001
		Finishing pigs	74		0.254 (−0.061 to 0.569)	0.114	93.6%	<0.001
Drip loss (%)	PUFA source	CLA	163	0.828	−0.147 (−0.314 to 0.021)	0.086	0.0%	0.857
		Linseed	105		−0.299 (−0.959 to 0.361)	0.374	81.9%	<0.001
	Concentration	High concentration	147	0.891	−0.268 (−0.775 to 0.240)	0.301	74.9%	<0.001
		Low concentration	121		−0.128 (−0.307 to 0.050)	0.159	0.0%	0.878
	Initial growth stage	Growing pigs	170	0.868	−0.135 (−0.301 to 0.031)	0.111	0.0%	0.777
		Finishing pigs	98		−0.258 (-0.955 to 0.440)	0.469	81.6%	<0.001
L*	PUFA source	CLA	205	0.226	−0.155 (−0.590 to 0.280)	0.485	14.0%	0.287
		Linseed	67		−1.740 (−3.267 to −0.213)	0.026	81.9%	<0.001
	Concentration	High concentration	65	0.932	−1.366 (−2.717 to −0.015)	0.047	78.9%	<0.001
		Low concentration	207		−0.172 (−0.627 to 0.283)	0.459	17.4%	0.254
	Initial growth stage	Growing pigs	182	0.266	−0.091 (−0.652 to 0.470)	0.750	34.5%	0.092
		Finishing pigs	90		−1.331 (−2.354 to −0.308)	0.011	72.5%	<0.001
a*	PUFA source	CLA	205	0.500	0.137 (−0.265 to 0.538)	0.504	73.3%	<0.001
		Linseed	67		−0.066 (−0.578 to 0.446)	0.800	36.8%	0.148
	Concentration	High concentration	65	0.269	0.146 (−0.364 to 0.656)	0.574	54.7%	0.024
		Low concentration	207		0.053 (−0.370 to 0.476)	0.806	73.0%	<0.001
	Initial growth stage	Growing pigs	182	0.179	0.227 (−0.141 to 0.595)	0.226	61.7%	0.001
		Finishing pigs	90		−0.255 (−0.900 to 0.391)	0.439	75.8%	<0.001
b*	PUFA source	CLA	205	0.187	−0.194 (−0.344 to −0.044)	0.011	52.7%	0.005
		Linseed	67		0.184 (−0.140 to 0.508)	0.265	26.7%	0.225
	Concentration	High concentration	65	0.890	0.032 (−0.209 to 0.273)	0.794	17.1%	0.290
		Low concentration	207		−0.180 (−0.351 to −0.009)	0.039	60.3%	0.001
	Initial growth stage	Growing pigs	182	0.686	−0.163 (−0.295 to −0.030)	0.016	32.0%	0.112
		Finishing pigs	90		−0.104 (−0.475 to 0.267)	0.583	70.0%	<0.001
pH 45 min	PUFA source	CLA	108	0.772	0.019 (−0.095 to 0.132)	0.749	64.1%	0.003
		Linseed	105		0.056 (−0.059 to 0.172)	0.337	77.7%	0.002
	Concentration	High concentration	93	0.774	0.058 (−0.064 to 0.179)	0.352	79.9%	<0.001
		Low concentration	120		0.021 (−0.086 to 0.128)	0.704	60.6%	0.003
	Initial growth stage	Growing pigs	118	0.185	−0.003 (−0.078 to 0.072)	0.940	31.6%	0.123
		Finishing pigs	95		0.106 (−0.054 to 0.266)	0.195	87.5%	<0.001
pH 24 h	PUFA source	CLA	195	0.742	−0.006 (−0.023 to 0.11)	0.486	0.0%	0.568
		Linseed	87		−0.034 (−0.049 to −0.018)	<0.001	1.2%	0.420
	Concentration	High concentration	85	0.673	−0.033 (−0.049 to −0.018)	<0.001	0.0%	0.526
		Low concentration	197		−0.006 (−0.023 to 0.011)	0.479	0.0%	0.486
	Initial growth stage	Growing pigs	172	0.108	−0.002 (−0.019 to 0.015)	0.813	0.0%	0.783
		Finishing pigs	110		−0.035 (−0.051 to −0.020)	<0.001	0.7%	0.434

a *N, total number of pigs*.

^b^P_regression_, P value of regression, significance level P_regression_ < 0.05. L*, lightness; a*, redness; b*, yellowness.

### Effects of CLA and Linseed Supplementation on the Meat Quality and Growth Performance of Pigs

**Figure 2:**
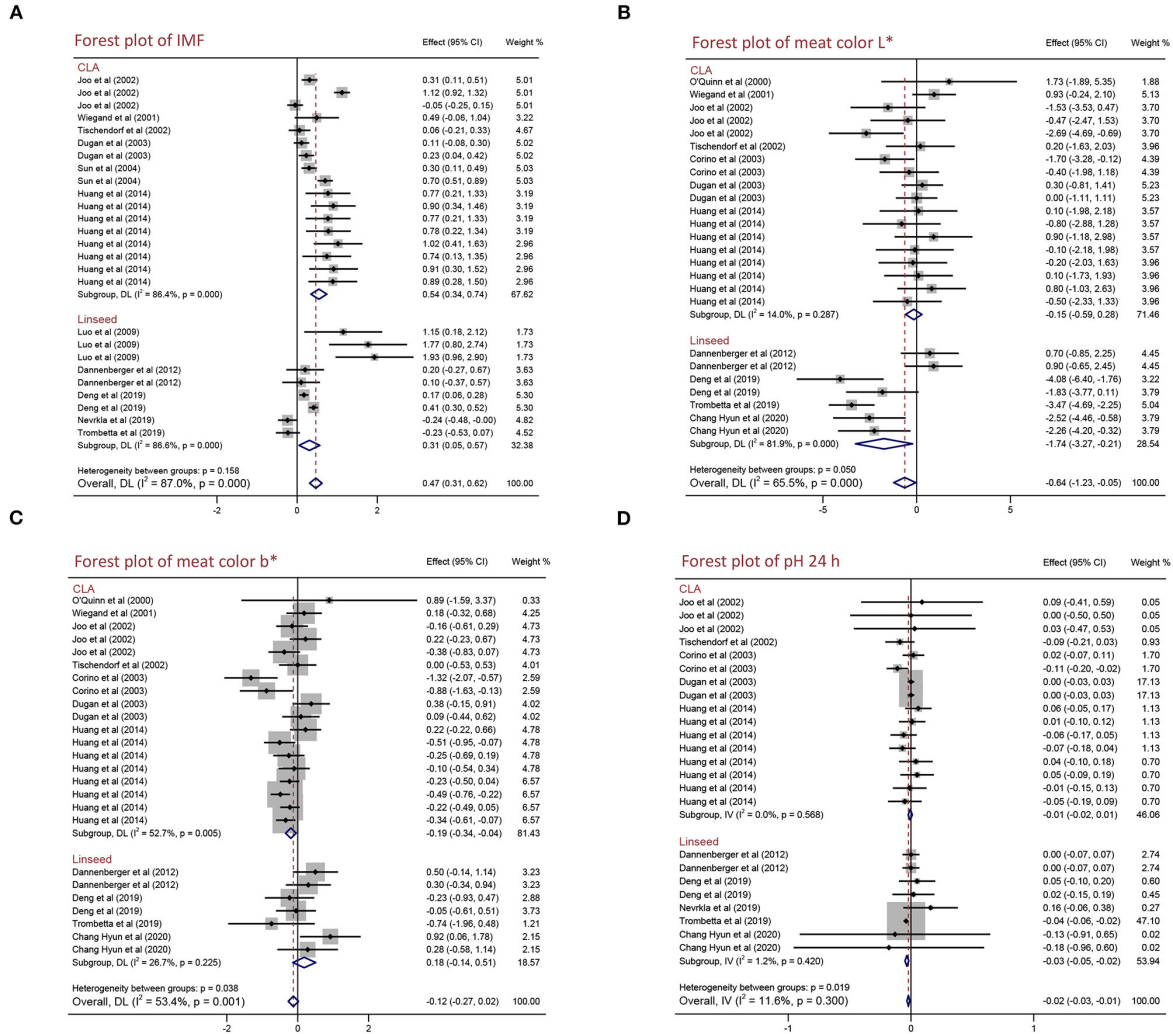
Forest plot of the effects of CLA or linseed on the meat quality of pigs. **(A)** IMF, **(B)** meat color L*, **(C)** meat color b*, and **(D)** pH 24 h. WMD, weighted mean difference; CLA, conjugated linoleic acid. The small solid diamond represents the point estimate for each individual trial, and the horizontal line extending from each solid diamond represents the upper and lower limits of the 95% CI. The size of the shaded square indicates the relative weight of the trial in the meta-analysis. The hollow diamond represents the WMD and 95% CI of the trials, no intersection of the diamond and the solid black line in the middle indicates a significant difference (*p* < 0.05), vice versa.

To explain the effects of CLA and linseed supplementation on the meat quality and growth performance of pigs, we performed a subgroup analysis of different PUFA source [CLA and linseed (linseed or linseed oil)]. As shown in Figure 2 and Table 4, both CLA and linseed supplementation increased IMF content by 0.542% (95% CI: 0.343–0.741, *p* < 0.001) with high heterogeneity (*I*^2^ = 86.4%, *p*_heterogeneity_ < 0.001) and 0.307% (95% CI: 0.047–0.566, *p* = 0.021) with high heterogeneity (*I*^2^ = 86.6%, *p*_heterogeneity_ < 0.001). CLA supplementation can decrease meat color b^*^ by 0.194 (95% CI: −0.344 to −0.044, *p* = 0.011) with moderate heterogeneity (*I*^2^ = 52.7%, *p*_heterogeneity_ = 0.005). However, there are no effects on drip loss, meat color L^*^, meat color a^*^, pH 45 min, and pH 24 h (*p* > 0.05) (Table 4). Linseed and linseed oil decreased meat color L^*^ (WMD = −1.740, 95% CI: −3.267 to −0.213, *p* = 0.026) with high heterogeneity (*I*^2^ = 81.9%, *p*_heterogeneity_ < 0.001) and pH 24 h (WMD = −0.034, 95% CI: −0.049 to −0.018, *p* < 0.001) with no heterogeneity (*I*^2^ = 1.2%, *p*_heterogeneity_ = 0.420). To sum up, CLA supplementation increased IMF content and decreased meat color b^*^, whereas linseed supplementation reduced meat color L^*^ and pH 24 h. Furthermore, we found no significant differences in other meat quality and growth performance indexes (*p* > 0.05) (Table 4 and Supplementary Table 4).

### Effects of Different PUFA Supplementation Concentration on the Meat Quality and Growth Performance of Pigs

**Figure 3:**
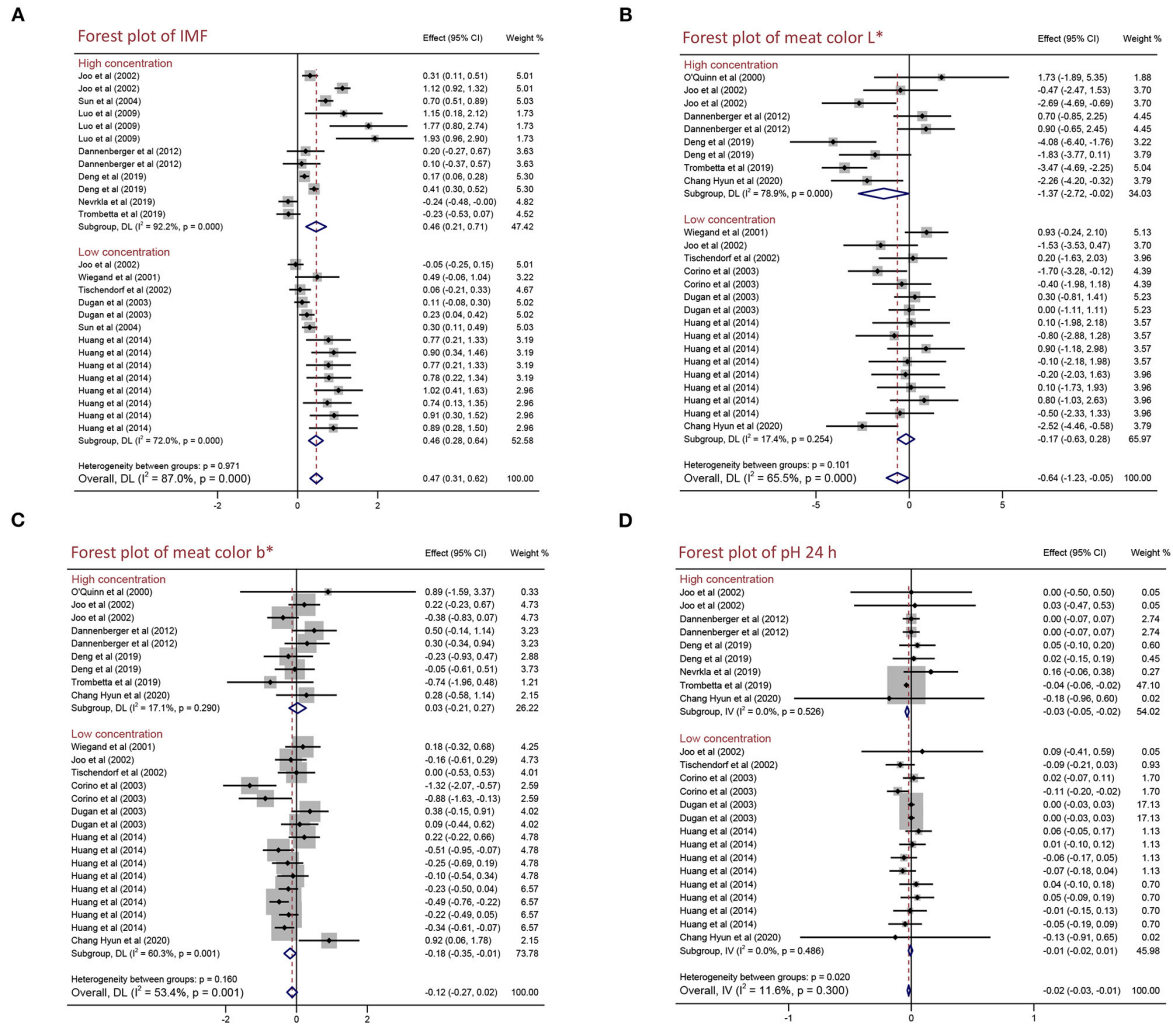
Forest plot of the differences in the meat quality of pigs fed high/low PUFA supplementation concentration. **(A)** IMF, **(B)** meat color L*, **(C)** meat color b*, and **(D)** pH 24 h. WMD, weighted mean difference; CLA, conjugated linoleic acid. The small solid diamond represents the point estimate for each individual trial, and the horizontal line extending from each solid diamond represents the upper and lower limits of the 95% CI. The size of the shaded square indicates the relative weight of the trial in the meta-analysis. The hollow diamond represents the WMD and 95% CI of the trials, no intersection of the diamond and the solid black line in the middle indicates a significant difference (*p* < 0.05), vice versa.

To explore the effects of different PUFA supplementation concentration on the meat quality and growth performance of pigs, we performed a subgroup analysis of different concentration [high concentration (>2%) and low concentration (≤ 2%)]. As presented in Figure 3 and Table 4, not only high concentration but also low concentration improved IMF content by 0.461% (95% CI: 0.208–0.715, *p* < 0.001) and 0.456% (95% CI: 0.276–0.635, *p* < 0.001) with high heterogeneity (*I*^2^ = 92.2%, *p*_heterogeneity_ < 0.001) and moderate heterogeneity (*I*^2^ = 72.0%, *p*_heterogeneity_ < 0.001). High PUFA supplementation concentration decreased meat color L^*^ (WMD = −1.366, 95% CI: −2.717 to −0.015, *p* = 0.047) with high heterogeneity (*I*^2^ = 78.9%, *p*_heterogeneity_ < 0.001) and pH 24 h (WMD = −0.033, 95% CI: −0.049 to −0.018, *p* < 0.001) with no heterogeneity (*I*^2^ = 0.0%, *p*_heterogeneity_ = 0.526). Additionally, we found low concentration reduced meat color b^*^ by 0.180 (95% CI: −0.351 to −0.009, *p* = 0.039) with moderate heterogeneity (*I*^2^ = 60.3%, *p*_heterogeneity_ = 0.001) but increased ADFI by 50.000 g/day (95% CI: 49.957–50.043, *p* < 0.001) with no heterogeneity (*I*^2^ = 0.0%, *p*_heterogeneity_ = 0.768). In conclusion, high PUFA supplementation concentration improved IMF content and reduced meat color L^*^ and pH 24 h, whereas low concentration decreased meat color b^*^ and increased ADFI. There are no significant differences on other indexes (drip loss, meat color a^*^, pH 45 min, ADG, and G:F ratio; *p* > 0.05) (Table 4 and Supplementary Table 4).

### Effects of PUFA Supplementation on the Meat Quality and Growth Performance of Growing and Finishing Pigs

**Figure 4:**
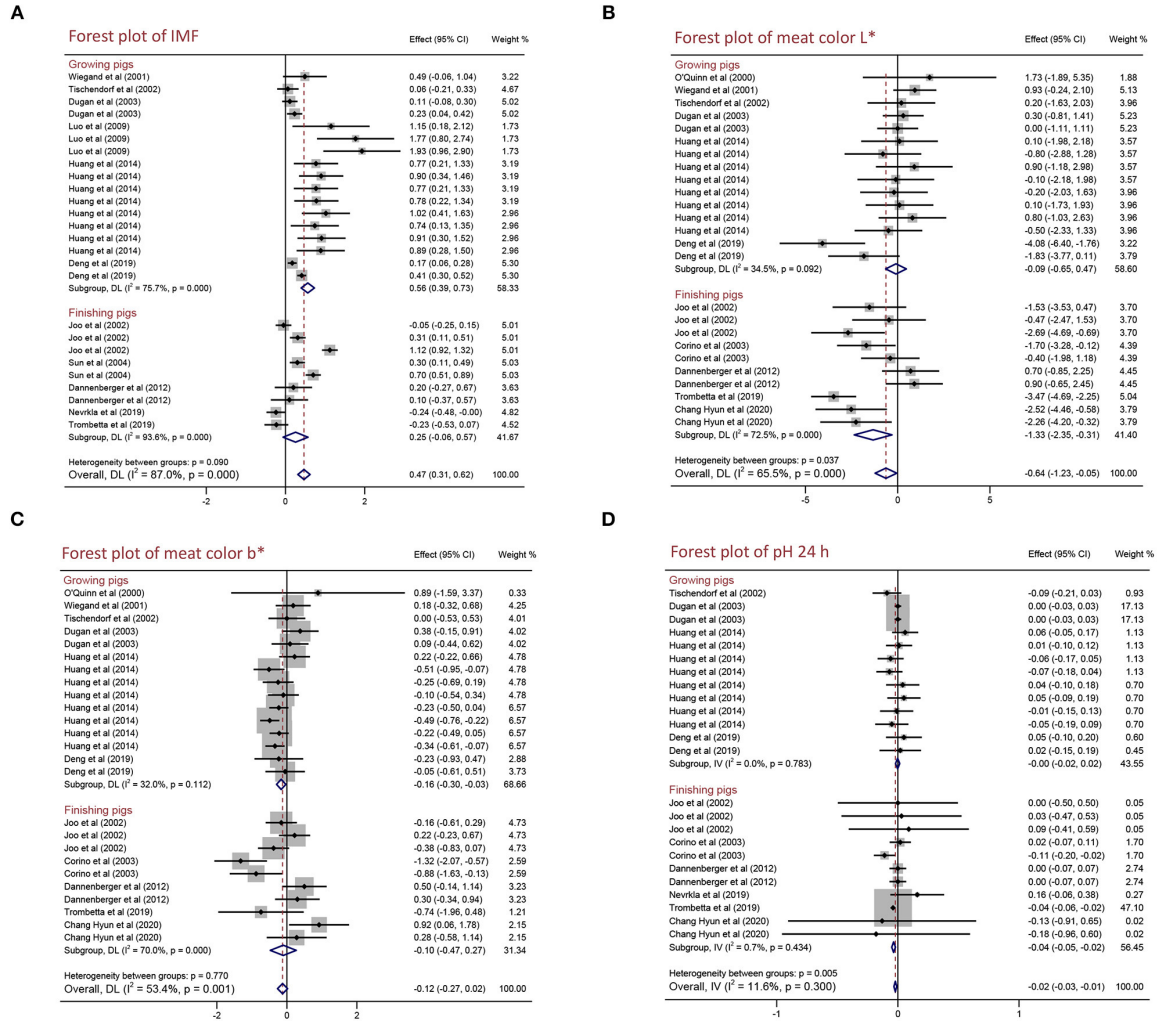
Forest plot of the effects of polyunsaturated fatty acid (PUFA) supplementation on the meat quality of growing and finishing pigs. **(A)** IMF, **(B)** meat color L*, **(C)** meat color b*, and **(D)** pH 24 h. WMD, weighted mean difference; CLA, conjugated linoleic acid. The small solid diamond represents the point estimate for each individual trial, and the horizontal line extending from each solid diamond represents the upper and lower limits of the 95% CI. The size of the shaded square indicates the relative weight of the trial in the meta-analysis. The hollow diamond represents the WMD and 95% CI of the trials, no intersection of the diamond and the solid black line in the middle indicates a significant difference (*p* < 0.05), vice versa.

As shown in Figure 4 and Table 4, for growing pigs, PUFA supplementation increased IMF content by 0.563% (95% CI: 0.395–0.731, *p* < 0.001) with high heterogeneity (*I*^2^ = 75.7%, *p*_heterogeneity_ < 0.001), whereas decreased meat color b^*^ by 0. 163 (95% CI: −0.295 to −0.030, *p* = 0.016) with low heterogeneity (*I*^2^ = 32.0%, *p*_heterogeneity_ = 0.112). Moreover, PUFA supplementation reduced meat color L^*^ by 1.331 (95% CI: −2.354 to −0.308, *p* = 0.011) with moderate heterogeneity (*I*^2^ = 72.5%, *p*_heterogeneity_ < 0.001), pH 24 h by 0.035 (95% CI: −0.051 to −0.020, *p* < 0.001) with no heterogeneity (*I*^2^ = 0.7%, *p*_heterogeneity_ = 0.434), and improved ADFI by 103.847 g/day (95% CI: −36.922 to 170.772, *p* < 0.002) with high heterogeneity (*I*^2^ = 100.0%, *p*_heterogeneity_ < 0.001) in finishing pigs. In a word, PUFA supplementation increased IMF content and decreased meat color b^*^ in growing pigs, whereas reduced meat color L^*^, pH 24 h but improved ADFI in finishing pigs. Additionally, we found PUFA supplementation had no influence on drip loss, meat color a^*^, pH 45 min, ADG, and G:F ratio in both growing pigs and finishing pigs (*p* > 0.05) (Table 4 and Supplementary Table 4).

## Discussion

Pork is one of the most popular meats among people because of its rich nutrition, good flavor, and good economic benefits, and pork has been the first meat for many years. However, in pig production, meat quality has been declining in recent years due to the blind pursuit of production efficiency and increase of backfat thickness. The evaluation index of pork quality includes meat color, tenderness, pH, flavor, IMF, drip loss, and so on (31, 32). Pork quality is affected by many factors, such as genetics (breed), nutrition sex, and environment (feeding management conditions, pre-slaughter conditions, etc.). Nutritional intervention is one of the most common methods to improve pork quality. Recent studies have found that PUFA supplementation in diet had an effect on the meat quality of pigs, but the results are inconsistent. Our meta-analysis demonstrated that PUFA supplementation can significantly increase IMF content but decrease meat color L^*^ and pH 24 h in pigs (Table 3). Furthermore, we also found there is a little energy level difference between control and treatment groups in dietary diet. Hence, the effects of different diets on meat quality mainly are due to the PUFA supplementation rather than energy level. Overall, these data support that PUFA supplementation is a benefit for improving meat quality in pigs.

It has been reported that the content of IMF is positively related to pork quality including tenderness, flavor, and juiciness (33). How to improve IMF content is one of the most urgent problems in the pig industry. IMF is mainly distributed in the epimysium, perimysium, and endomysium of skeletal muscle in which the main components are phosphoric acid and triglyceride. Previous studies found that IMF content is related to breed, sex, diet, and weight at slaughter in pigs (34–36). PUFAs are one of the essential fatty acids and play an important role in regulating fat deposition, muscle development, and glycolipid metabolism (7, 8). CLA is a group of positional and geometric isomers of linoleic acid with a conjugated double bond, which is generally found in ruminant animals and dairy products and has many physiological functions including anti-obesity, anti-diabetic, anti-cancer, and anti-hypertension (37). Linseed is the ripe seed of flax, which is rich in n-3 PUFAs and has anti-obesity, anti-inflammatory, anti-cancer, and regulating glucose and lipid metabolism effects (38). In our meta-analysis, we observed that the dietary PUFA supplementation can increase IMF content, not only CLA but also linseed supplementation significantly improved IMF content (Figure 2A). The concentration of PUFA supplementation in diet might influence the effects on pork quality. However, we found the benefit of PUFA supplementation on IMF content is not dependent on concentration and PUFA supplementation has positive effects in different breeds (foreign and Chinese local pigs) (Figure 3A and Supplementary Table 5). Hence, PUFA supplementation can be a nutritional measure to regulate IMF content. However, only growing pigs had an increased IMF content after being fed PUFA supplementation, and finishing pigs had an insignificant effect (Figure 4A). It might because there are nutritional requirements (energy, amino acids, minerals, etc.) that differ in pigs at different growth stages.

Meat color and pH are some of the most important factors that affect the sensory quality of pork (39, 40). However, current studies on the effects of PUFA supplementation on meat color and pH are controversial. Meat color will turn bright red into dark red when pork is placed for a certain time. The difference in myoglobin content influences meat color, and the ratio of the three forms of myoglobin (deoxy myoglobin, oxygen myoglobin, and ferric myoglobin) determine meat color (41, 42). Currently, people use a flesh-color meter to determine the color of meat, including lightness (L^*^), redness (a^*^), and yellowness (b^*^). In our analysis, dietary PUFA supplementation significantly decreased L^*^, but a^*^ and b^*^ were not influenced (Table 3). We conjectured it might be because PUFA supplementation led to myoglobin oxidation and decreased the ratio of oxygen myoglobin to reduce lightness but had no influence on redness and yellowness. We also discovered that the effects of PUFA supplementation on L^*^ and b^*^ are dependent on concentration, growth stage, and breeds (Figures 3, 4 and Supplementary Table 5). The pH value is an important index to reflect the muscle contraction and glycolysis rate of pigs after slaughter. After slaughter, pH decreased rapidly from 7.0–7.2 to 5.5–6.5, which was mainly due to muscle glycolysis and lactic acid production. Furthermore, the alteration of meat color and pH results from the different post-mortem processes are affected by environmental factors, such as nutrition, breeding conditions, transport conditions, stress, weather conditions, and the methods of slaughter (43). A previous study summarized those differences in pork quality including flavor, tenderness, odor, and acidity resulted from the environment of delivery, the feeding environment (temperature, humidity, breeding density, etc.), and the pre-slaughter environment (excessive stress and excessively hungry before slaughter) (39). Additionally, some genetic genes are also reported to affect meat color and pH value following influence on pork quality. The recessive homozygote of *halothane* gene causes stress in pigs and reduces pork quality, and the adverse allele of *rendement napole* gene can significantly increase muscle glycogen content, produce more lactic acid, and decrease pH value (44). Particularly, pigs are subjected to excessive stress and severe hypoxia before slaughter, and a large amount of lactic acid was produced by glycolysis in the body lead to low pH 45 min value (below 5.5), which is often associated with pale meat color, resulting in pale, soft and exudative (PSE) pork. In contrast, pigs are excessively hungry before slaughter and a large amount of glycogen in the body is used for energy, resulting in insufficient glycogen in the body after slaughter lead to high meat pH 24 h (above 6.4) often causes dark, firm, and dry (DFD) pork (45). Post-slaughter glycolysis produces lactic acid and reduces the pH of meat, and the speed and duration of the post-slaughter glycolysis determine the development of PSE, DFD, or normal meat. Our results showed PUFA supplementation significantly decreased pH 24 h but did not affect pH 45 min (Table 3). Due to the pH 45 min is an indicator of the speed of the glycolysis and pH 24 h is the consequence of the whole period glycolysis, we assume PUFAs might affect muscle contraction and production of lactic acid but not affect glycolysis rate after slaughter. Even though CLA and linseed are all PUFAs, they had different effects on L^*^, b^*^, and pH 24 h (Figure 2), and it might result from different fatty acids composition. Furthermore, we demonstrated that PUFA supplementation significantly decreased pH 24 h in foreign pigs but not significantly reduced in Chinese local pigs (Supplementary Table 5) because different breeds have different nutritional requirements. In addition, we found neither pH 24 h values above 6.4 nor pH 45 min below 5.5 in any studies, and there is no significant effect on growth performance parameters including ADG, ADFI, and G:F ratio in pigs. Hence, PUFA supplementation might provide a safe and useful strategy to improve pork quality.

As shown in Table 4, the significant heterogeneity in the drip loss and L^*^of pigs was primarily driven by the linseed, high concentration, and finishing pigs subgroup. Differently, the CLA, low concentration, and finishing pigs subgroup are sources of b^*^ heterogeneity. CLA and finishing pigs subgroup are the source of a^*^ and pH 45 min heterogeneity, respectively. Additionally, we found ADG and ADFI had high heterogeneity. Although we demonstrated that the significant heterogeneity in ADFI is driven by the high concentration group, we still thought the high heterogeneity was due to the number of included studies for growth performance analysis was too small. Because we did not find the source of IMF heterogeneity according to the subgroup analysis, we performed a sensitivity analysis by using the leave-one-out method on IMF. However, the significant heterogeneity had no change after each included study was removed; thus, we assume that the meta-analysis results are robust, and the heterogeneity did not influence the significance of pooled estimates. Furthermore, we used a fixed-effects model to analyze pH 24 h and G:F ratio due to the homogeneity (*I*^2^ < 50%).

A limitation of this meta-analysis is that the effects of PUFA supplementation duration and actual grams of PUFA intake on meat quality of pigs and whether PUFA supplementation could affect sex of pigs are unknown as a result of the incomplete data, and we assume that further studies should focus on these questions. Furthermore, as SD values are important for meta-analysis and they affect many estimates, including the weight of an individual study, the 95% CI, and heterogeneity, so the lack of within-group SD might influence the results of the meta-analysis. We used pooled SD as within-group SD, and it might be impacted by the number of groups and SEM. To verify our findings are reliable, we checked the consistency between 95% CI pooled estimate and the significance and tendency of included studies. Hence, our results are valid, and this method is appropriate for analyzing non-ruminant studies, which lack within-group SD. Furthermore, there is another method that can be used for estimating within-group SD, which is suitable for studies that reported the median, range, and size of a sample (46). In a word, different approaches can be adopted to estimate within-group SD and accordingly ensure the results of the meta-analysis are reliable and robust.

## Conclusions

Our results indicate that PUFA supplementation increases IMF content, decreases meat color L^*^ and pH 24 h but has no effect on other meat quality and growth performance indexes in pigs; this result is related to PUFA supplementation in the diet rather than the energy level in the basal diet. Our systemic analysis suggests that PUFA supplementation has beneficial influences on improving the meat quality of pigs, which mainly emerges in increasing IMF content in finishing pigs without considering breeds. Hence, we assume that using PUFA supplementation (both CLA and linseed have a function) in the diet is a safe and useful strategy to improve pork quality and without concentration- and breed-dependent to get the best results in increasing IMF content according to our meta-analysis. This may become an effective method for producing high-quality pork in the pig industry, but the optimal PUFA supplementation concentration needs to be further studied.

## Data Availability Statement

The original contributions presented in the study are included in the article/Supplementary Material, further inquiries can be directed to the corresponding author/s.

## Author Contributions

LW and YH participated in study quality assessment and study criteria selection. LW extracted data, conducted the statistical analysis, and wrote the final version of the manuscript. YH checked the data and assisted in the interpretation and revising of the article. YW and TS oversaw the development of the study and resolved conflicts in the meta-analysis. All authors have read and approved the final manuscript.

## Funding

The project was partially supported by the Key Research and Development Program of Zhejiang Province (2021C02008), the Zaozhuang Talent Program Funding, the “Hundred Talents Program” funding from Zhejiang University to TS.

## Conflict of Interest

The authors declare that the research was conducted in the absence of any commercial or financial relationships that could be construed as a potential conflict of interest.

## Publisher's Note

All claims expressed in this article are solely those of the authors and do not necessarily represent those of their affiliated organizations, or those of the publisher, the editors and the reviewers. Any product that may be evaluated in this article, or claim that may be made by its manufacturer, is not guaranteed or endorsed by the publisher.
