# Supplementary material for: Effects of Polyunsaturated Fatty Acids Supplementation on the Meat Quality of Pigs: A Meta-Analysis
Source: Front Nutr. 2021 Sep 29;8:746765. doi: 10.3389/fnut.2021.746765 (PMC8511515; doi:10.3389/fnut.2021.746765)
Supplement: Supplementary file 1 [file Data_Sheet_1.docx]

***Supplementary Material***

- 1. **Supplementary Tables**

**Table S1.** The statistical growth performance parameters and outcomes of included studies ^1^.

| **Study** | **Growth stage** | **PUFA source** | **Concentration** | **ADG(g/d)^2^** | | **ADFI(g/d)^3^** | | **G:F ratio^4^** | | **IMF (%)^5^** | | **Drip loss (%)** | | **Meat color ^6^** | | | | | | **pH 45 min ^7^** | | **pH 24 h ^8^** | | |
| --- | --- | --- | --- | --- | --- | --- | --- | --- | --- | --- | --- | --- | --- | --- | --- | --- | --- | --- | --- | --- | --- | --- | --- | --- |
|  |  |  |  |  |  |  |  |  |  |  |  |  |  | **L** | | **a** | | **b** | |  |  |  |  |  |
|  |  |  |  | **T** | **C** | **T** | **C** | **T** | **C** | **T** | **C** | **T** | **C** | **T** | **C** | **T** | **C** | **T** | **C** | **T** | **C** | **T** | **C** |  |
| O'Quinn et al., 2000 | Growing pigs | Conjugated linoleic acid | 50% | 970 | 1030 | 2780 | 2920 | 0.35 | 0.35 | NA | NA | 2.83 | 3.03 | 52.66 | 50.93 | 12.01 | 10.80 | 7.89 | 7.00 | NA | NA | NA | NA |  |
| Wiegand et al., 2001 | Growing pigs | Conjugated linoleic acid | 0.75% | 950 | 940 | 2714.3 | 2764.7 | 0.35 | 0.34 | 3.04* | 2.55 | NA | NA | 50.43* | 49.50 | 5.80 | 5.48 | 11.59 | 11.41 | NA | NA | NA | NA |  |
| Joo et al., 2002 | Finishing pigs | Conjugated linoleic acid | 1% | NA | NA | NA | NA | NA | NA | 2.48 | 2.53 | 4.87 | 5.13 | 45.66 | 47.19 | 7.29 | 6.24 | 4.44 | 4.60 | NA | NA | 5.63 | 5.54 |  |
| Joo et al., 2002 | Finishing pigs | Conjugated linoleic acid | 2.5% | NA | NA | NA | NA | NA | NA | 2.84 | 2.53 | 5.06 | 5.13 | 46.72 | 47.19 | 7.36 | 6.24 | 4.82 | 4.60 | NA | NA | 5.54 | 5.54 |  |
| Joo et al., 2002 | Finishing pigs | Conjugated linoleic acid | 5% | NA | NA | NA | NA | NA | NA | 3.65* | 2.53 | 4.38 | 5.13 | 44.50 | 47.19 | 6.25 | 6.24 | 4.22 | 4.60 | NA | NA | 5.57 | 5.54 |  |
| Tischendorf et al., 2002 | Growing pigs | Conjugated linoleic acid | 2% | 701 | 687 | 2040 | 1990 | 0.34 | 0.35 | 1.54 | 1.48 | 6.1 | 5.7 | 53.1 | 52.9 | 7.2 | 7.2 | 4.5 | 4.5 | 5.79 | 5.82 | 5.37 | 5.46 |  |
| Corino et al., 2003 | Finishing pigs | Conjugated linoleic acid | 0.25% | NA | NA | NA | NA | NA | NA | NA | NA | NA | NA | 43.6 | 45.3 | 16.09 | 18.39 | 12.36 | 13.68 | 6.01 | 5.74 | 5.82 | 5.80 |  |
| Corino et al., 2003 | Finishing pigs | Conjugated linoleic acid | 0.5% | NA | NA | NA | NA | NA | NA | NA | NA | NA | NA | 44.9 | 45.3 | 16.71 | 18.39 | 12.80 | 13.68 | 5.94 | 5.74 | 5.69 | 5.80 |  |
| Dugan et al., 2003 | Growing pigs | Conjugated linoleic acid | 0.25% | NA | NA | NA | NA | NA | NA | 1.93 | 1.82 | 5.96 | 5.87 | 53.9 | 53.6 | 8.78 | 8.45 | 5.52 | 5.14 | NA | NA | 5.54 | 5.54 |  |
| Dugan et al., 2003 | Growing pigs | Conjugated linoleic acid | 0.5% | NA | NA | NA | NA | NA | NA | 2.05* | 1.82 | 5.87 | 5.87 | 53.6 | 53.6 | 8.62 | 8.45 | 5.23 | 5.14 | NA | NA | 5.54 | 5.54 |  |
| Sun et al., 2004 | Finishing pigs | Conjugated linoleic acid | 2% | 840** | 780 | 2610** | 2510 | 0.32** | 0.31 | 2.7 | 2.4 | 4.9 | 5.2 | NA | NA | NA | NA | NA | NA | NA | NA | NA | NA |  |
| Sun et al., 2004 | Finishing pigs | Conjugated linoleic acid | 4% | 890** | 780 | 2700** | 2510 | 0.33** | 0.31 | 3.1** | 2.4 | 4.9 | 5.2 | NA | NA | NA | NA | NA | NA | NA | NA | NA | NA |  |
| Luo et al., 2009 | Growing pigs | Linseed | 30 d (10%) | NA | NA | NA | NA | NA | NA | 3.64 | 2.49 | 5.01 | 5.19 | NA | NA | NA | NA | NA | NA | 6.37 | 6.47 | NA | NA |  |
| Luo et al., 2009 | Growing pigs | Linseed | 60 d (10%) | NA | NA | NA | NA | NA | NA | 4.26* | 2.49 | 4.83 | 5.19 | NA | NA | NA | NA | NA | NA | 6.57 | 6.47 | NA | NA |  |
| Luo et al., 2009 | Growing pigs | Linseed | 90 d (10%) | NA | NA | NA | NA | NA | NA | 4.42* | 2.49 | 3.63 | 5.19 | NA | NA | NA | NA | NA | NA | 6.58 | 6.47 | NA | NA |  |
| Dannenberger et al., 2012 | Finishing pigs | Linseed oil | High (4.5%) | 835.2* | 761.0 | 3195.6 | 3214.2 | 0.26 | 0.24 | 1.4 | 1.2 | 2.6 | 4.6 | 48.3 | 47.6 | 7.4 | 7.7 | 1.6 | 1.1 | 6.4 | 6.2 | 5.4 | 5.4 |  |
| Dannenberger et al., 2012 | Finishing pigs | Linseed oil | Reduced (4.5%) | 840.6* | 761.0 | 3195.0 | 3214.2 | 0.26 | 0.24 | 1.3 | 1.2 | 3.0* | 4.6 | 48.5 | 47.6 | 7.3 | 7.7 | 1.4 | 1.1 | 6.4 | 6.2 | 5.4 | 5.4 |  |
| Huang et al., 2014 | Growing pigs | Conjugated linoleic acid | 0.5% (30-60 kg) | NA | NA | NA | NA | NA | NA | 2.49* | 1.72 | NA | NA | 34.8 | 34.7 | 4.66 | 4.62 | 3.06 | 2.84 | 6.94 | 6.84 | 5.89 | 5.83 |  |
| Huang et al., 2014 | Growing pigs | Conjugated linoleic acid | 1% (30-60 kg) | NA | NA | NA | NA | NA | NA | 2.62* | 1.72 | NA | NA | 33.9 | 34.7 | 4.30 | 4.62 | 2.33* | 2.84 | 6.53* | 6.84 | 5.84 | 5.83 |  |
| Huang et al., 2014 | Growing pigs | Conjugated linoleic acid | 1.5% (30-60 kg) | NA | NA | NA | NA | NA | NA | 2.49* | 1.72 | NA | NA | 35.6 | 34.7 | 3.96 | 4.62 | 2.59 | 2.84 | 6.68 | 6.84 | 5.77 | 5.83 |  |
| Huang et al., 2014 | Growing pigs | Conjugated linoleic acid | 2% (30-60 kg) | NA | NA | NA | NA | NA | NA | 2.50* | 1.72 | NA | NA | 34.6 | 34.7 | 4.46 | 4.62 | 2.74 | 2.84 | 6.48* | 6.84 | 5.76 | 5.83 |  |
| Huang et al., 2014 | Finishing pigs | Conjugated linoleic acid | 0.5% (60-90 kg) | NA | NA | NA | NA | NA | NA | 3.21* | 2.19 | 2.11 | 2.02 | 34.0 | 34.2 | 4.50 | 4.49 | 2.66 | 2.89 | 6.38 | 6.29 | 5.42 | 5.38 |  |
| Huang et al., 2014 | Finishing pigs | Conjugated linoleic acid | 1% (60-90 kg) | NA | NA | NA | NA | NA | NA | 2.93* | 2.19 | 1.78 | 2.02 | 34.3 | 34.2 | 4.29 | 4.49 | 2.40* | 2.89 | 6.34 | 6.29 | 5.43 | 5.38 |  |
| Huang et al., 2014 | Finishing pigs | Conjugated linoleic acid | 1.5% (60-90 kg) | NA | NA | NA | NA | NA | NA | 3.10* | 2.19 | 1.70 | 2.02 | 35.0 | 34.2 | 4.68 | 4.49 | 2.67 | 2.89 | 6.42 | 6.29 | 5.37 | 5.38 |  |
| Huang et al., 2014 | Finishing pigs | Conjugated linoleic acid | 2% (60-90 kg) | NA | NA | NA | NA | NA | NA | 3.08* | 2.19 | 1.98 | 2.02 | 33.7 | 34.2 | 5.12 | 4.49 | 2.55* | 2.89 | 6.35 | 6.29 | 5.33 | 5.38 |  |
| Deng et al., 2019 | Growing pigs | Flaxseed | 5% | 782.96 | 752.22 | 2370.95 | 2514.70 | 0.33 | 0.30 | 1.93* | 1.76 | 3.02 | 3.26 | 55.21* | 59.29 | 17.97 | 17.34 | 3.81 | 4.04 | 6.23 | 6.10 | 5.83 | 5.78 |  |
| Deng et al., 2019 | Growing pigs | Flaxseed | 10% | 787.24 | 752.22 | 2420.98 | 2514.70 | 0.33 | 0.30 | 2.17* | 1.76 | 3.25 | 3.26 | 57.46 | 59.29 | 18.10 | 17.34 | 3.99 | 4.04 | 6.14 | 6.10 | 5.80 | 5.78 |  |
| Nevrkla et al., 2019 | Finishing pigs | Linseed | 7% | NA | NA | NA | NA | NA | NA | 2.48* | 2.72 | 5.79*** | 4.87 | NA | NA | NA | NA | NA | NA | 5.89** | 5.77 | 5.67** | 5.51 |  |
| Trombetta et al., 2019 | Finishing pigs | Linseed oil | 3% | 840 | 850 | NA | NA | NA | NA | 7.16 | 7.39 | 10.66 | 7.42 | 47.01 | 50.48 | 10.80 | 12.10 | 11.30 | 12.04 | NA | NA | 5.30 | 5.34 |  |
| Chang Hyun et al., 2020 | Finishing pigs | Linseed | 4:1(1.5%) | 757* | 695 | 2562 | 2482 | 0.29 | 0.28 | NA | NA | 6.17 | 6.42 | 49.39 | 51.91 | 7.25 | 7.26 | 6.98 | 6.06 | 6.17 | 6.13 | 5.42 | 5.55 |  |
| Chang Hyun et al., 2020 | Finishing pigs | Linseed | 2:1(3%) | 768* | 695 | 2714 | 2482 | 0.28 | 0.28 | NA | NA | 5.65 | 6.42 | 49.65 | 51.91 | 7.44 | 7.26 | 6.34 | 6.06 | 6.14 | 6.13 | 5.37 | 5.55 |  |

^1^ T, treatment, C, control; NA, not available; a significant difference in the trial is indicated by *P < 0.05 and **P < 0.01.

^2^ ADG, average daily gain.

^3^ ADFI, average daily feed intake.

^4^ G: F ratio, gain: feed ratio.

^5^ IMF, intramuscular fat content.

^6^ L, lightness, a, redness, b, yellowness.

^7^ pH 45 min, pH value measured at 45 min postmortem.

^8^ pH 24 h, pH value measured at 24-hour postmortem.

**Table S2.** Study quality assessment.

| **Study** | **Within-group differences ^1^** | **Multiple reports ^2^** | **Sample size ^3^** | **Rationality of experimental design** | **Completeness of experimental information** | **Score ^4^** | **Quality** |
| --- | --- | --- | --- | --- | --- | --- | --- |
| Chang Hyun et al., 2020 | 0 | 5 | 4 | 3 | 4 | 16 | Moderate |
| Corino et al., 2003 | 0 | 5 | 2 | 5 | 3 | 15 | Moderate |
| Dannenberger et al., 2012 | 5 | 5 | 1 | 4 | 5 | 20 | Moderate |
| Deng et al., 2019 | 5 | 5 | 3 | 3 | 5 | 21 | High |
| Dugan et al., 2003 | 0 | 5 | 4 | 3 | 5 | 17 | Moderate |
| Huang et al., 2014 | 0 | 5 | 5 | 3 | 4 | 17 | Moderate |
| Joo et al., 2002 | 0 | 5 | 1 | 5 | 4 | 15 | Moderate |
| Luo et al., 2009 | 0 | 5 | 1 | 4 | 3 | 13 | Low |
| Nevrkla et al, 2019 | 5 | 5 | 2 | 4 | 4 | 20 | Moderate |
| O'Quinn et al., 2000 | 0 | 5 | 1 | 4 | 2 | 12 | Low |
| Sun et al., 2004 | 0 | 5 | 2 | 4 | 2 | 15 | Low |
| Tischendorf et al., 2002 | 5 | 5 | 2 | 4 | 5 | 21 | High |
| Trombetta et al., 2019 | 0 | 5 | 1 | 4 | 4 | 14 | Low |
| Wiegand et al., 2001 | 0 | 5 | 1 | 3 | 2 | 11 | Low |

^1^ Within-group differences: Within-group SD/SE not reported (sub-score = 0); Within-group SD/SE reported (sub-score = 5).

^2^ Multiple reports: The same batch of pigs was reported in several articles (sub-score = 0); No exist of multiple reports (sub-score = 5).

^3^ Sample size: >150 (sub-score= 5); 100-149 (sub-score = 4); 70-99 (sub-score = 3); 30-69 (sub-score = 2); <30 (sub-score = 1).

^4^ Score is the sum of 5 sub-scores. Score of quality: > 20 (High); 15-20 (Moderate); < 15 (Low).

**Table S3**. The summary of meta-analysis and publication bias analysis of growth performance in included studies

| **Outcome ^1^** | **N ^2^** | **WMD (95% CI) ^3^** | ***P*** | ***I^2^*** | ***P*_heterogeneity_** | **Begg's Test** | **Egger's test** |
| --- | --- | --- | --- | --- | --- | --- | --- |
| ADG (g/d) | 12 | -597.967 (-2.4e+03 to 1187.682) | 0.512 | 100.0% | <0.001 | 0.005 | 0.766 |
| ADFI (g/d) | 10 | 12.741 (-43.348 to 68.829) | 0.656 | 100.0% | <0.001 | 0.024 | 0.803 |
| G:F ratio | 9 | 0.010 (-0.002 to 0.023) | 0.108 | 0.0% | 0.986 | 1.000 | 0.885 |

^1^ ADG, average daily gain; ADFI, average daily feed intake; G: F ratio, gain: feed ratio.

^2^ N, number of comparisons.

^3^ WMD, weighted mean difference; CI, confidence interval.

**Table S4**. Regression and subgroup analysis of growth performance in included studies

| **Outcome^1^** | **Subgroup** | | **N^2^** | ***P*** **_regression_^3^** | **WMD (95% CI)** | ***P*** | ***I^2^*** | ***P* _heterogeneity_** |
| --- | --- | --- | --- | --- | --- | --- | --- | --- |
| ADG (g/d) | PUFA source | CLA | 98 | 0.642 | 26.913 (-38.827 to 92.654) | 0.422 | 100.0% | <0.001 |
|  |  | Linseed | 147 |  | -1.0e+03 (-5.1e+03 to 1996.054) | 0.612 | 100.0% | <0.001 |
|  | Concentration | High concentration | 159 | 0.850 | -806.845 (-2.9e+03 to 1270.036) | 0.414 | 100.0% | <0.001 |
|  |  | Low concentration | 86 |  | 25.765 (-3.578 to 55.108) | 0.085 | 79.6% | 0.007 |
|  | Initial growth stage | Growing pigs | 110 | 0.664 | 5.142 (-39.198 to 49.482) | 0.820 | 100.0% | <0.001 |
|  |  | Finishing pigs | 135 |  | -1.0e+03 (-3.7e+03 to 1659.917) | 0.453 | 100.0% | <0.001 |
| ADFI (g/d) | PUFA source | CLA | 88 | 0.249 | 50.000 (-27.235 to 127.235) | 0.205 | 100.0% | <0.001 |
|  |  | Linseed | 136 |  | -28.933 (-110.376 to 52.510) | 0.486 | 69.1% | 0.006 |
|  | Concentration | High concentration | 148 | 0.201 | 3.039 (-63.366 to 69.445) | 0.929 | 100.0% | <0.001 |
|  |  | Low concentration | 76 |  | 50.000 (49.957 to 50.043) | <0.001 | 0.0% | 0.768 |
|  | Initial growth stage | Growing pigs | 100 | 0.010 | -80.386 (-213.954 to 53.183) | 0.238 | 100.0% | <0.001 |
|  |  | Finishing pigs | 124 |  | 103.847 (-36.922 to 170.772) | 0.002 | 100.0% | <0.001 |
| G:F ratio | PUFA source | CLA | 98 | 0.818 | 0.013 (-0.005 to 0.030) | 0.157 | 0.0% | 0.923 |
|  |  | Linseed | 120 |  | 0.008 (-0.011 to 0.026) | 0.400 | 0.0% | 0.807 |
|  | Concentration | High concentration | 132 | 0.825 | 0.007 (-0.052 to 0.066) | 0.814 | 0.0% | 0.983 |
|  |  | Low concentration | 86 |  | 0.010 (-0.001 to 0.022) | 0.083 | 0.0% | 0.702 |
|  | Initial growth stage | Growing pigs | 110 | 0.928 | 0.011 (-0.028 to 0.050) | 0.565 | 0.0% | 0.916 |
|  |  | Finishing pigs | 108 |  | 0.010 (-0.003 to 0.024) | 0.133 | 0.0% | 0.787 |

^1^ ADG, average daily gain; ADFI, average daily feed intake; G: F ratio, gain: feed ratio.

^2^ N, total number of pigs.

^3^ *P* _regression_, *P* value of regression, significance level *P* _regression_ < 0.05.

**Table S5.** Subgroup analysis of studies included in the meta-analysis

| **Outcome** | **Subgroup^1^** | | **WMD (95% CI)** | ***P*** | ***I^2^*** | ***P*_heterogeneity_** |
| --- | --- | --- | --- | --- | --- | --- |
| IMF (%) | Breeds | Foreign | 0.420 (0.136 to 0.705) | 0.004 | 92.1% | <0.001 |
|  |  | Local | 0.844 (0.638 to 1.050) | <0.001 | 0.0% | 0.998 |
| Drip loss (%) | Breeds | Foreign | -0.289 (-0.824 to 0.245) | 0.288 | 74.8% | <0.001 |
|  |  | Local | -0.127(-0.323 to 0.068) | 0.202 | 0.0% | 0.455 |
| L* | Breeds | Foreign | -1.066(-2.024to -0.107)) | 0.029 | 71.0% | <0.001 |
|  |  | Local | 0.039 (-0.648 to 0.726) | 0.911 | 0.0% | 0.936 |
| a* | Breeds | Foreign | 0.098 (-0.664 to 0.861) | 0.800 | 82.0% | <0.001 |
|  |  | Local | -0.089 (-0.349 to 0.171) | 0.502 | 5.7% | 0.386 |
| b* | Breeds | Foreign | -0.076 (-0.400 to 0.248) | 0.645 | 64.1% | 0.001 |
|  |  | Local | -0.268 (-.406 to -0.130) | <0.001 | 25.4% | 0.227 |
| pH 45min | Breeds | Foreign | 0.077 (-0.037 to 0.190) | 0.187 | 81.1% | <0.001 |
|  |  | Local | -0.043 (-0.176 to 0.090) | 0.527 | 52.6% | 0.039 |
| pH 24h | Breeds | Foreign | -0.036 (-0.051 to -0.021) | <0.001 | 0.0% | 0.455 |
|  |  | Local | -0.006 (-0.049 to 0.036) | 0.767 | 0.0% | 0.622 |

^1^ Foreign, foreign pigs; Local, Chinese local pigs.

**1.2 Supplementary Figure**


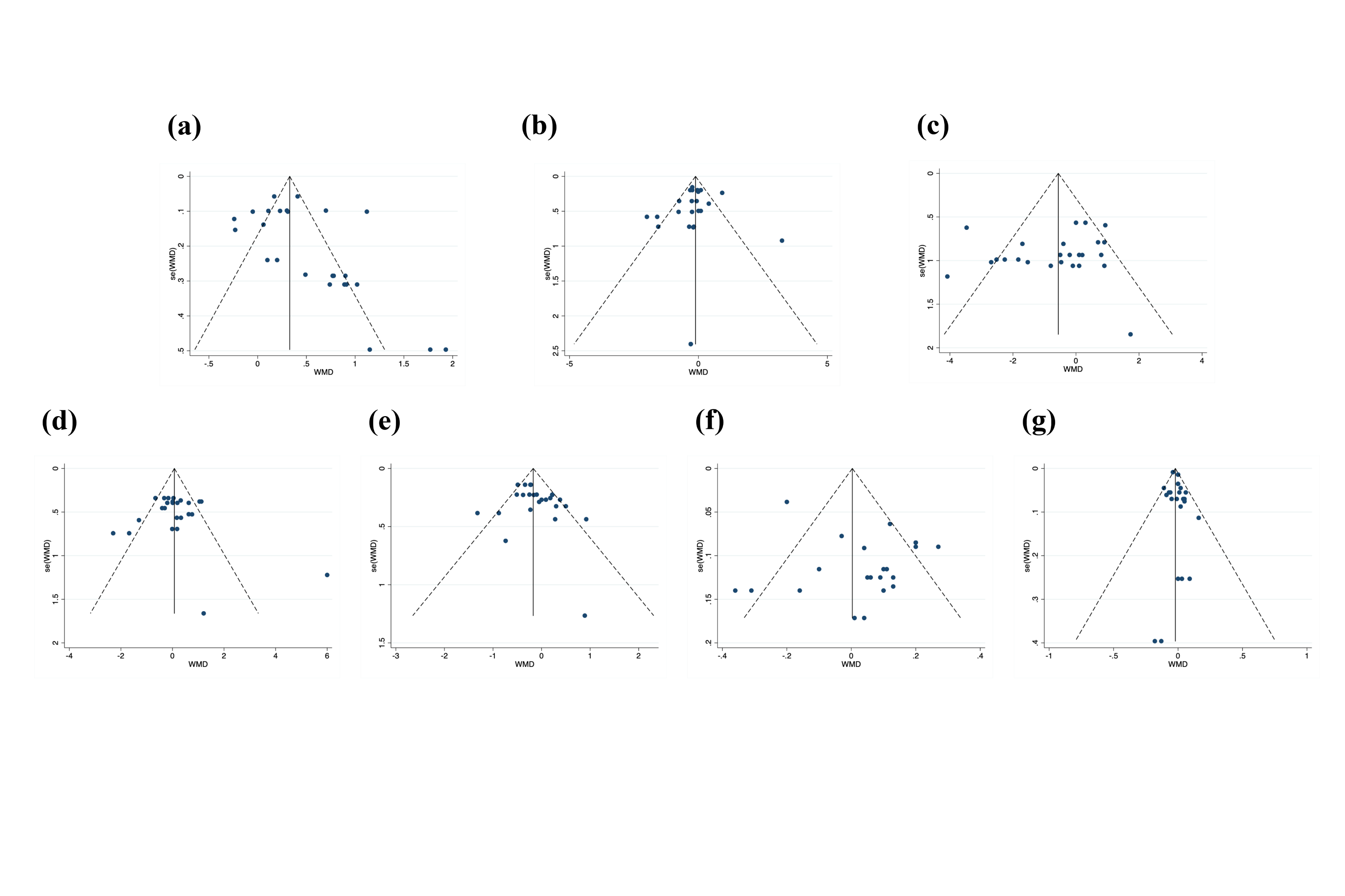


**Supplementary Figure 1.** Funnel plots. a IMF. b drip loss. c meat color L*. d meat color a*. e meat color b*. f pH 45 min. g pH 24 h.
